# Pilot study of a rapid and minimally instrumented sputum sample preparation method for molecular diagnosis of tuberculosis

**DOI:** 10.1038/srep19541

**Published:** 2016-01-20

**Authors:** Tanya M. Ferguson, Kris M. Weigel, Annie Lakey Becker, Delia Ontengco, Masahiro Narita, Ilya Tolstorukov, Robert Doebler, Gerard A. Cangelosi, Angelika Niemz

**Affiliations:** 1 Claremont BioSolutions, LLC, Upland, CA; 2 University of Washington, Department of Environmental and Occupational Health Sciences, Seattle, WA; 3 Seattle Biomedical Research Institute, Seattle, WA; 4 University of Santo Tomas Graduate School, Manila, Philippines; 5 Keck Graduate Institute of Applied Life Sciences, Claremont, CA; 6 Public Health - Seattle & King County, TB Control Program, Seattle, WA.

## Abstract

Nucleic acid amplification testing (NAAT) enables rapid and sensitive diagnosis of tuberculosis (TB), which facilitates treatment and mitigates transmission. Nucleic acid extraction from sputum constitutes the greatest technical challenge in TB NAAT for near-patient settings. This report presents preliminary data for a semi-automated sample processing method, wherein sputum is disinfected and liquefied, followed by PureLyse^®^ mechanical lysis and solid-phase nucleic acid extraction in a miniaturized, battery-operated bead blender. Sputum liquefaction and disinfection enabled a >10^4^ fold reduction in viable load of cultured *Mycobacterium tuberculosis* (*M.tb*) spiked into human sputum, which mitigates biohazard concerns. Sample preparation via the PureLyse^®^ method and a clinically validated manual method enabled positive PCR-based detection for sputum spiked with 10^4^ and 10^5^ colony forming units (cfu)/mL *M.tb*. At 10^3^ cfu/mL sputum, four of six and two of six samples amplified using the comparator and PureLyse^®^ method, respectively. For clinical specimens from TB cases and controls, the two methods provided 100% concordant results for samples with 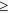
1 mL input volume (N = 41). The semi-automated PureLyse^®^ method therefore performed similarly to a validated manual comparator method, but is faster, minimally instrumented, and can be integrated into TB molecular diagnostic platforms designed for near-patient low-resource settings.

Tuberculosis (TB) is a major health threat worldwide with an estimated 9 million incident active TB cases and 1.5 million TB-associated deaths in 2013^1^, mainly in resource-limited settings within high-burden countries in Africa, Asia, and Eastern Europe. Despite international efforts^2^, active TB continues to be significantly under-diagnosed, or is diagnosed long after becoming symptomatic and infectious, which compromises the ability to effectively treat patients and curb transmission^3^,^4^,^5^.

In most endemic regions, diagnosis of active pulmonary TB relies on smear microscopy, which is relatively simple and low cost, but suffers from insufficient sensitivity and specificity^6^,^7^. Culture-based methods, the current gold standard for TB diagnosis, are highly sensitive but expensive, require biosafety level 3 (BSL3) laboratory infrastructure, and the time to result is usually several weeks. As a result, neither microscopy nor culture is sufficient for effective case finding, patient management, and containment of TB transmission.

Nucleic acid amplification testing (NAAT) is becoming more integral to TB diagnostics in developed and developing countries^8^. NAAT enables sensitive and specific TB diagnosis^4^,^9^,^10^, can identify drug resistance mutations, and in principle can provide results during the same patient visit, which reduces loss to follow-up. However, worldwide implementation of TB NAAT is hampered by its relatively high cost and complexity^8^. TB NAATs in use or in the development pipeline vary in complexity^11^ and entail either laboratory-developed tests^12^,^13^,^14^, or commercial systems^9^,^15^,^16^,^17^,^18^,^19^ based on the polymerase chain reaction (PCR)^9^,^12^,^19^,^20^,^21^, or isothermal amplification methods, such as loop-mediated isothermal amplification (LAMP)^15^,^22^, transcription-mediated amplification (TMA)^18^, cross-priming amplification (CPA)^16^, or helicase-dependent amplification (HDA)^17^,^23^.

The GeneXpert MTB/RIF is the most widely used TB NAAT system, following WHO endorsement for TB diagnosis and rifampin resistance testing, and global roll-out starting in 2011^24^. This highly automated platform performs nucleic acid sample preparation and hemi-nested PCR amplification with real-time detection^25^,^26^, with all reagents on board. The GeneXpert is recommended for use in district and sub-district level laboratories of countries where TB and MDR-TB are prevalent. However, the instrument and consumables cost remains substantial, and the infrastructure required cannot be accommodated in low-resource microscopy centers and primary care settings that serve the majority of the affected patient population. Several other NAAT systems are in late-stage development or on the market for TB diagnosis^8^,^15^,^16^. However, these systems lack integration of sample preparation with amplification and detection in a fully automated format, posing implementation challenges in low-resource, remote primary care settings. Methods with cumbersome and lengthy multi-step processes are error-prone, require skilled users, and in most cases additional consumables and laboratory instrumentation (e.g. centrifuges and heat baths) which may not be readily available^27^. Furthermore, low-resource settings often do not have uninterrupted power, which is required to operate such instruments^7^. The integration of sample preparation therefore is a significant challenge and bottleneck in enabling TB NAAT in near-patient low-resource settings. Minimally instrumented, fully enclosed, and battery-operated sample preparation technologies with a small footprint are ideal for such settings.

Many current TB NAAT methods process sputum specimens analogous to culture-based protocols^26^. First, raw sputum is liquefied, usually using N-acetyl-L-cysteine (NALC) and/or NaOH, to enable manipulation of the viscous matrix. Culture-based TB diagnosis requires sample decontamination, which preserves at least some live mycobacteria but inactivates other microorganisms which would otherwise overgrow the slow-growing mycobacteria. However, to protect the operator in low-resource settings with limited biosafety precautions, it is preferable for TB NAAT to disinfect the sputum, i.e. to render all pathogens including mycobacteria non-viable. Several reagents have been reported to effectively render *Mycobacterium tuberculosis* (*M.tb*) non-viable including sodium hypochlorite, povidone iodine, and hydrogen peroxide^28^,^29^, or in the case of the GeneXpert MTB/RIF assay, isopropanol with NaOH^26^. For some TB NAAT methods, the liquefied sputum is centrifuged to pellet bacteria^12^,^13^,^22^, and the pellet is then washed and re-suspended, to remove impurities and in some cases concentrate the bacteria in a smaller volume^30^. This approach resembles culture- and microscopy-based TB diagnostic methods, but centrifugation is difficult to automate. In the GeneXpert MTB/RIF assay, bacteria in the processed sputum are separated and concentrated through automated filtration and washing^9^,^25^.

Mycobacteria have thick waxy cell envelopes that are resistant to many conventional chemical or enzymatic lysis methods. Lysis of mycobacteria in most cases entails either heating the sample to ≥80 °C for 20 min to 1 h^12^,^31^, or mechanical disruption through sonication^25^,^32^,^33^ or high-energy bead beating^32^,^34^. If mycobacteria have been isolated from sputum prior to lysis, the lysed material is typically used directly for nucleic acid amplification. Alternatively, if mycobacteria are lysed in the sputum sample, the DNA can be purified by removing inhibitors e.g. through adsorption to a zeolite matrix^15^, through standard solid-phase extraction methods^13^,^14^ or sequence-specific capture^23^,^35^.

Claremont BioSolutions (ClaremontBio) developed a miniaturized, battery-operated bead beating system for mechanical pathogen lysis, called the OmniLyse^®^ device. In previous work, this device was shown to effectively disrupt tough-walled microorganisms, such as *M.tb* complex bacteria and *Bacillus subtilis* spores and liberate their nucleic acids in a format suitable for PCR amplification^34^. ClaremontBio’s miniaturized bead beating system (Fig. 1) can also perform solid-phase DNA extraction, using the PureLyse^®^ technology^36^, which does not require chaotropic salts or organic solvents that can inhibit downstream polymerase amplification^37^,^38^.

This report describes a novel nucleic acid sample preparation method from sputum which can be coupled with PCR to detect *M.tb* genomic DNA. The method incorporates sample disinfection and liquefaction, followed by mechanical lysis and solid-phase extraction of liberated nucleic acids using the PureLyse^®^ technology. This semi-automated approach is compared to a clinically validated manual sample preparation method of sputum liquefaction, isolation of bacteria via centrifugation, and heat lysis to liberate nucleic acids, developed by the Wadsworth Center at the New York State Department of Health^12^. The method described herein can be completed in <20 min, much faster than the comparator method, uses disposable battery-operated components, protects users by disinfecting samples at the outset, and is suitable for automation. In ongoing efforts, we are integrating this method with DNA amplification and detection in a disposable cartridge and portable battery-operated instrument^39^,^40^, which has the potential to facilitate near-patient diagnosis of TB in resource-limited settings.

## Results

We developed a sputum disinfection and liquefaction method, based on trisodium phosphate (TSP) as liquefaction reagent^33^ and povidone iodine (PVI) as disinfectant^28^,^29^. Various formulations of these components were explored, along with necessary incubation times to achieve sample liquefaction and mycobactericidal properties. We conducted a kill study to quantify the effectiveness of the protocol at inactivating *M.tb* complex cells in sputum, as described in the Methods section. Sputum samples spiked with *M.tb* H37Rv were treated using the disinfection/liquefaction protocol, and replicates of undiluted and 10-fold diluted samples were plated. Treated spiked sputum samples contained a few *M.tb* colonies (<10) on some plates streaked with undiluted sample, but no colonies were observed for the diluted samples (Table 1). Based on control experiments in buffer without disinfection, each spiked sample contained >10^6^ cfu/mL *M.tb* H37Rv in the final suspension, of which >10^5^ cfu *M.tb* H37Rv were plated for the undiluted samples. Therefore, we obtained a >4-log reduction in viability relative to these controls. Furthermore, non-mycobacterial colonies observed in unprocessed sputum controls were not observed in the disinfected samples, suggesting a broad microbicidal effect.

**Figure 1:**
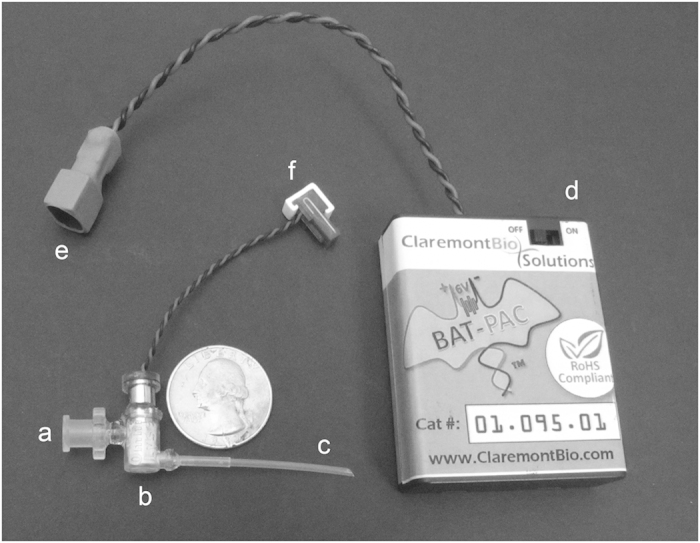
Disposable miniaturized battery-operated PureLyse^®^ bead blender for mechanical cell lysis and solid-phase nucleic acid extraction. Sample in binding buffer, wash, and elution buffer are introduced at the sample inlet (**a**) and flow through the PureLyse^®^ chamber (**b**) containing beads, micro-motor, and impeller, exiting via the outlet (**c**). Battery pack (Bat-Pac) (**d**) connected to blender motor via connectors (**e, f**). Printed with permission from Claremont BioSolutions.

In conjunction with this sputum disinfection and liquefaction method, a rapid sputum sample preparation method was employed, using ClaremontBio’s PureLyse^®^ system to lyse *M.tb* and extract nucleic acids in a miniaturized and minimally instrumented format, with a 3-step protocol that takes less than 10 minutes to complete. The PureLyse^®^ cartridge (Fig. 1) contains a micro-motor equipped with a precision-cut impeller capable of operating at up to 30,000 rpm with power supplied by a 6 V battery pack. The cartridge is packed with beads to generate shear forces sufficient for mechanical lysis of tough-walled organisms, and to bind and release DNA under specific buffer conditions which enables solid-phase nucleic acid extraction^34^,^36^.

**Figure 2:**
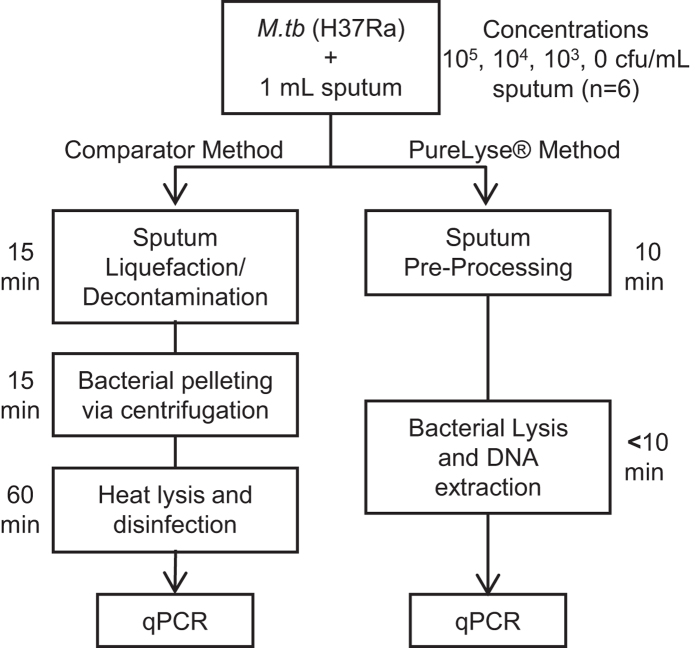
Schematic diagram of the experimental design comparing the comparator^12^ and PureLyse® sample preparation methods. TB-negative pooled sputum (~1 mL) samples were spiked with 10^5^, 10^4^, 10^3^, or 0 cfu *M.tb* H37Ra and processed by both methods. Extracted nucleic acids were then amplified and detected using qPCR, as described by Halse *et al.*^12^.

The PureLyse^®^ protocol (liquefaction, disinfection, and nucleic acid extraction) was compared to an established and clinically validated protocol for nucleic acid extraction from sputum for molecular TB diagnosis, developed by Halse *et al.*^12^. For initial analytical validation, cultured *M.tb* H37Ra was spiked into *M.tb-*negative human sputum at three concentrations and processed using the PureLyse^®^ and comparator sample preparation methods. Nucleic acid from samples purified by each method were amplified and detected via the qPCR protocol of Halse *et al.* (Fig. 2)^12^.

**Figure 3:**
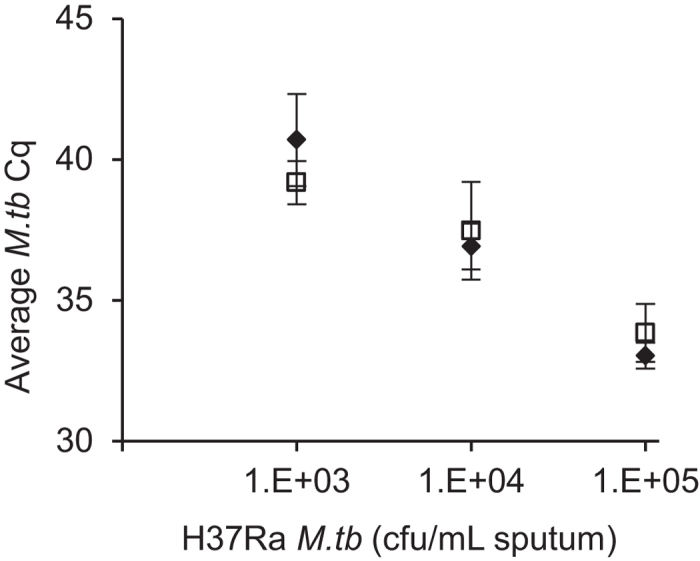
qPCR detection of *M.tb* H37Ra spiked into sputum, extracted via the comparator (black diamond) and PureLyse^®^ methods (white square): mean and standard deviation from six biological replicates, with two technical replicates each. At 10^5^ and 10^4^ cfu/mL, all replicate samples amplified. At 10^3^ cfu/mL, four of six samples and two of six samples amplified for the comparator and PureLyse^®^ methods, respectively.

The PureLyse^®^ and comparator sample preparation methods performed comparably for sputum samples spiked with 10^4^ and 10^5^ cfu/mL H37Ra (Fig. 3). At these two concentrations, 100% of the samples (N = 6) amplified by both methods, with comparable Cq values [no statistically significant difference, p = 0.283 (10^4^ cfu/mL) and p = 0.054 (10^5^ cfu/mL), paired sample T-test, 2-tailed]. At 10^3^ cfu/mL, four of six samples amplified using the comparator method whereas two of six samples amplified using the PureLyse^®^ method. A sample’s extract was considered positive if at least one of two qPCR technical replicates yielded a positive result. We observed no substantial inhibition for either extraction method, as the average internal amplification control Cq’s were comparable for samples processed via the PureLyse^®^ method (30.37 ± 0.31), comparator method (29.99 ± 0.24), and for the DNA standards and no template controls (29.83 ± 0.15), with no statistically significant difference (p = 0.0564 (PureLyse^®^), p = 0.328 (comparator), One-way ANOVA]. None of the negative controls showed false amplification.

To test the performance of the PureLyse^®^ protocol on clinical sputum specimens from TB positive and negative patients, a similar experimental approach was used to analyze 46 sputum specimens collected from patients of the Seattle-King County Tuberculosis Control Clinic. Smear and culture results were not available for each of these individual specimens. Smear and culture analysis was only performed on three initial samples used for laboratory diagnosis, which were collected before the study samples were obtained. Some of the study samples from patients with active TB were therefore expected to be negative for *M.tb* DNA by the Purelyse® and comparator methods due to normal fluctuations in bacillary load. Of the 30 specimens obtained from TB positive subjects, 24 samples were positive and five samples were negative following sample preparation by the comparator method. One sample was excluded from further analysis due to a technical error during processing. All 16 specimens from TB-negative subjects gave negative qPCR results using both methods.

Of the 24 samples determined to be positive based on the comparator method, 22 yielded positive qPCR results following PureLyse^®^ sample preparation, while two samples yielded negative qPCR results. Both of the PureLyse^®^ method “false negatives” had sample input volumes <1 mL (a deviation from the standard protocol) and also gave weak signals after extraction via the comparator method. Overall, this pilot clinical study resulted in a true positive fraction (sensitivity relative to the comparator method) of 91.7% (95% Confidence Interval (CI): 71.5–98.5%) when considering all samples (Table 2, “All Samples”), or 100% (95% CI: 80–100%) when considering only samples with input volume >1mL (Table 2, “Samples of ≥1 mL volume”). The true negative fraction (specificity) relative to the comparator method was 100% in both cases (95% CI: 80.8–100%). Comparable average internal amplification control Cq values were observed for the PureLyse^®^ method (28.33 ± 0.42), comparator method (28.04 ± 0.78), and for the DNA standards and no template controls (28.25 ± 0.34), which indicates negligible inhibition for either sample extraction method.

## Discussion

This report presents preliminary analytical and clinical studies, comparing semi-automated PureLyse^®^ sputum sample preparation to an established and clinically validated manual sample preparation method described by Halse *et al.*^12^. In the analytical evaluation, the comparator method performed slightly better for sputum spiked with *M.tb* H37Ra at 10^3^ cfu/mL, the lowest concentration tested, while identical performance was observed at 10^4^ and 10^5^ cfu/mL. Both methods gave concordant results when performed on patient-derived clinical samples, except when the sputum sample input volume was less than 1 mL, for which some samples did not amplify following PureLyse^®^ sample preparation, and amplified late following the comparator sample preparation method. Since substantial inhibition was not observed using either method, discrepancies are likely linked to the DNA extraction yield. Ongoing optimization to improve the sensitivity of the PureLyse^®^-based sputum sample preparation method achieved promising results through refinements in disinfection and liquefaction composition, lysis and binding configuration, and buffer composition.

The PureLyse^®^ sputum sample preparation method described herein improves the occupational safety of DNA extraction from sputum for molecular diagnosis of TB. In addition to a >10^4^-fold reduction in *M.tb* viability, other microorganisms in sputum were rendered uncultivable on Middlebrook media after <10 minutes of treatment. In contrast, many sputum sample preparation protocols, including the comparator method, are not microbicidal until further downstream in the process. The GeneXpert MTB/RIF assay includes a sample disinfection step, enabling >10^6^ fold reduction in viable *M.tb* bacterial load^26^. Through further improvements to our current disinfection method, we anticipate that a similar efficacy can be obtained.

While there are limitations attributed to small data sets for both the analytical and clinical studies and further validation of the PureLyse^®^ extraction system is required following subsequent optimization efforts, the preliminary data demonstrate the potential utility in TB NAAT diagnostics. The PureLyse^®^ sputum sample preparation method is battery powered, minimally instrumented, and does not require additional equipment such as centrifuges and heat baths. In ongoing efforts, the PureLyse^®^ protocol and mechanical design are being incorporated into a cartridge to enable fully integrated sample preparation, isothermal amplification and lateral flow detection, controlled by a portable, inexpensive instrument^39^,^40^. Such devices could enable rapid TB diagnosis at the point-of-care in low-resource, high-burden areas globally.

## Methods

### Sample Sources and General Study Design

Analytical studies were performed using as matrix pooled human sputum purchased from BioreclamationIVT (Westbury, NY), confirmed by us to be negative for *M.tb* complex genomic DNA via the qPCR method of Halse *et al.*^12^. *M.tb* H37Ra (ATCC 25177, cultured in Difco Middlebrook 7H9 broth with 0.2% glycerol, 40 mM sodium pyruvate, 10% [v/v] ADC enrichment and 0.05% Tween 80) was spiked into TB-negative sputum to a final concentration of 10^3^, 10^4^, or 10^5^ cfu/mL. For each concentration, plus a TB-negative sputum control spiked with media only, six 1 mL aliquots were processed via the PureLyse^®^ and comparator sample preparation method,^12^ followed by amplification and detection using qPCR^12^, as described below.

Clinical sputum specimens were collected at the Public Health–Seattle & King County TB Clinic (Seattle, WA) from de-identified male and female subjects 18 years or older able to provide spontaneous sputum specimens. Protocols and consent forms were approved by Institutional Review Boards at the University of Washington and Claremont Graduate University. Informed consent was obtained from all subjects, and sample collection was carried out in accordance with the approved protocol. Up to ten specimens were collected per subject, with at least eight hours between each sample. For subjects with active TB, samples were collected within seven days of treatment initiation. Of the 46 specimens included in this study, 30 were obtained from 17 subjects subsequently diagnosed by microbiological culture and clinical criteria as active pulmonary TB cases; and 16 specimens came from ten patients who microbiologically and clinically did not have active TB. Each clinical sputum sample was divided into two aliquots of equal volume, typically ~1 mL, which were then processed respectively via the PureLyse^®^ and comparator sample preparation method^12^, followed by qPCR amplification and detection^12^. For five of the 46 samples, aliquots <1 mL (750–900 μL) were used due to insufficient total sample volume. One low-volume specimen was subsequently excluded from data analysis due to a technical problem during process execution.

### PureLyse® Sputum Nucleic Acid Sample Preparation Method

Samples were processed using the PureLyse^®^ kit (Claremont BioSolutions LLC), modified for sputum nucleic acid sample preparation to enable sputum liquefaction and disinfection, followed by miniaturized mechanical cell disruption and solid-phase nucleic acid extraction. *Sputum liquefaction and disinfection:* A disinfection/liquefaction reagent containing PVI (Rite Aid Corporation) and TSP was prepared within ≤2 min before use and vortexed within ≤10 sec before adding 0.334 mL to each ~1 mL sputum sample. Sample tubes were then vortexed briefly and incubated at room temperature for 10 minutes. *Nucleic acid extraction:* An equal volume of 2× binding buffer was added to the disinfected and liquefied sample, followed by vortexing. The sample was then pumped through the PureLyse^®^ blender using a programmable syringe pump (KD Scientific, KDS-250) at a flow rate of 1 mL/min, with the PureLyse^®^ motor activated, enabling cell lysis and DNA binding to the beads. Next, 4 mL wash buffer (0.2× binding buffer) was pumped through the PureLyse^®^ blender at a flow rate of 0.75 mL/min with the motor activated, followed by 1 mL of air purging, with the motor de-activated. To elute the nucleic acids from the beads, the PureLyse^®^ chamber was filled with ~150 μL elution buffer, the motor was activated for 30 seconds, and then the syringe pump was used to pump another ~150 μL elution buffer through the chamber at a flow rate of 0.33 mL/min. The final eluate was stored on ice for immediate downstream amplification by qPCR.

### Verification of Sample Disinfection

To evaluate the effectiveness of sputum sample disinfection, *M.tb* H37Rv (ATCC 25618, cultured in Difco Middlebrook 7H9 broth with 10% [v/v] ADC enrichment and 0.05% Tween 80) was spiked into pooled TB-negative human sputum to a final concentration of ~10^7^ cfu/mL (estimated by optical density), and 1 mL spiked sputum aliquots were processed using the sputum liquefaction and disinfection protocol. Disinfection was stopped after the 10 min incubation by diluting the sample to 50 mL with sterile phosphate buffered saline (PBS), followed by inversion and vortexing. Cells and other particulates were pelleted at 4000 × *g* for 20 minutes, supernatants were removed, and the pellets re-suspended in 1 mL PBS. Ten-fold dilution series of each cell suspension were prepared in PBS. Six replicate 100 μL aliquots (3 replicates each of 2 separate experiments) of undiluted and diluted samples were plated on Difco Middlebrook 7H10 agar with 10% (v/v) OADC enrichment, incubated at 37 °C for 6–8 weeks, then colonies were counted.

In parallel with the spiked sputum experiments, the number of *M.tb* H37Rv cells remaining after identical processing but in the absence of disinfection was determined microbiologically. These controls were designed to isolate the effect of disinfection from confounding causes of cell loss (e.g. adherence to sample tubes and pipette tips, aspiration, etc.) and thereby quantify the effect of disinfection alone. Sputum was not used as matrix since overgrowth with non-mycobacterial organisms present in sputum would confound the results. Here, *M.tb* H37Rv was spiked into 1 mL PBS aliquots, and processed as described above. Six replicate 100 μL aliquots (3 replicates each of 2 separate experiments) of undiluted and 10× diluted samples were plated and incubated, and colonies were counted. Six unprocessed sputum controls (unspiked and untreated) were also plated directly. After incubation for 6–8 weeks, these unprocessed sputum samples formed diverse colonies too numerous to count.

### Comparator Method for Sputum Nucleic Acid Sample Preparation

As a comparative nucleic acid extraction method, sputum samples were processed as described by Halse *et al.*^12^. Briefly, an equal volume of 3.5% NaOH was added to each ~1 mL sputum sample. Samples were vortexed and incubated for 15 min at room temperature to liquefy and decontaminate the sputum. Decontamination was stopped by diluting the samples with PBS (pH 6.8) to a final volume of 50 mL, followed by inversion mixing. The contents were pelleted by centrifugation (15 min, 3000 × *g*), and supernatants were carefully removed by aspiration. Pellets were resuspended in 1 mL PBS, heated at 80 °C for 1 hr to lyse the mycobacteria, and stored on ice until qPCR was performed.

### PCR Amplification and Detection

We used a qPCR method for detecting *M. tuberculosis* complex (MTBC) DNA, with internal amplification control, as described by Halse *et al.*^12^, with 0.8 mg/mL BSA added to the master-mix. This qPCR method targets the MTBC-specific insertion element *IS6110* and a sequence within an engineered internal control plasmid (pIC) flanked with identical primer binding sites. The IS6110 and pIC amplicons can be differentiated with FAM and VIC labeled sequence-specific hydrolysis probes. Each sample was analyzed in two separate reactions: one with only the *IS6110*-FAM probe, the other with the *IS6110-*FAM probe, the pIC-VIC probe, and 1 fg/μL pIC. The degree of PCR inhibition was assessed by comparing Cq values for the internal amplification control (pIC-VIC probe) relative to those in uninhibited reactions receiving water or purified genomic DNA as template. PCR amplification and detection was executed at two sites, using either a CFX96 (Bio-Rad) or StepOnePlus (Applied Biosystems).

## Additional Information

**How to cite this article**: Ferguson, T. M. *et al.* Pilot study of a rapid and minimally instrumented sputum sample preparation method for molecular diagnosis of tuberculosis. *Sci. Rep.* **6**, 19541; doi: 10.1038/srep19541 (2016).

## Figures and Tables

**Table 1:** Microbiological verification of sputum disinfection.

Run	Number of *M.tb* colonies after sputum disinfection				
	Sputum dilution				
	Replicate	undiluted	10^−1^	10^−2^	10^−3^
1^a^	A	1	0	0	0
	B	0	0	0	0
	C	1	0	0	0
2^b^	A	0	0	0	0
	B	8	0	0	0
	C	2	0	0	0

^a^Untreated control cell concentration 3 × 10^6^ cfu/mL;

^b^Untreated control cell concentration 1.7 × 10^6^ cfu/mL.

**Table 2:** Clinical laboratory evaluation of PureLyse^®^ sputum sample preparation.

All Samples		Comparator Method^b^		
			pos	neg
		45	24	21
PureLyse^®^ Method^a^	pos	22	22	0
	neg	23	2	21
**Samples of ≥1 mL volume**				
		41	20	21
PureLyse^®^ Method^a^	pos	20	20	0
	neg	21	0	21

^a^PureLyse^®^ sputum sample preparation of clinical sputum samples,

^b^sample preparation of clinical sputum samples using the comparator method described by Halse *et al.*^12^ followed by qPCR analysis.
